# Applying aspiration in local search for satisfiability

**DOI:** 10.1371/journal.pone.0231702

**Published:** 2020-04-23

**Authors:** Cong Peng, Zhongwei Xu, Meng Mei

**Affiliations:** 1 School of Electronic Information Engineering, Tongji University, Shanghai, China; 2 LIACS, Leiden University, Leiden, the Netherlands; Shandong University of Science and Technology, CHINA

## Abstract

The Boolean Satisfiability problem (SAT) is a prototypical NP-complete problem, which has been widely studied due to its significant importance in both theory and applications. Stochastic local search (SLS) algorithms are among the most efficient approximate methods available for solving certain types of SAT instances. The quantitative configuration checking (QCC) heuristic is an effective approach for improving SLS algorithms on solving the SAT problem, resulting in an efficient SLS solver for SAT named Swqcc. In this paper, we focus on combining the QCC heuristic with an aspiration mechanism, and then design a new heuristic called QCCA. On the top of Swqcc, we utilize the QCCA heuristic to develop a new SLS solver dubbed AspiSAT. Through extensive experiments, the results illustrate that, on random 3-SAT instances, the performance of AspiSAT is much better than that of Swqcc and Sparrow, which is an influential and efficient SLS solver for SAT. In addition, we further enhance the original clause weighting schemes employed in Swqcc and AspiSAT, and thus obtain two new SLS solvers called Ptwqcc and AspiPT, respectively. The eperimental results present that both Ptwqcc and AspiPT outperform Swqcc and AspiSAT on random 5-SAT instances, indicating that both QCC and QCCA heuristics are able to cooperate effectively with different clause weighting schemes.

## Introduction

The Boolean satisfiability (SAT) problem is one of the most studied NP-complete problems, and is of significant importance in both theory and pracite [1]. The SAT problem has a broad range of applications in various fields, such as mathematical logic, inference, machine learning, constraint satisfaction, VLSI engineering and computing theory [2]. Similarly, many real-world problems, including testing [3], formal verification [4, 5], synthesis [6], nano-fabric cell mapping [7] and various routing problems [8], can be encoded into SAT, and further be solved by efficient SAT solvers [9–12].

Given a propositional formula in conjunctive normal form (CNF), the SAT problem is to decide whether there exists an assignment of Boolean variables that makes the propositional formula evaluate to be true. Although it is important to solve the SAT instance encoded from industrial problems, solving random SAT instances, especially random 3-SAT instances, also shows great importance in both theory and practice. In theory, the random 3-SAT problem is the core problem in the field of computational complexity research. In practice, the random 3-SAT problem can provide a test platform for heuristic methods for dealing with many problems in machine learning and artificial intelligence [13]. Also, a random 3-SAT instance can provide a relatively unbiased and intractable test set for evaluating practical algorithms, making the results of practical algorithms statistically more significant. Due to the importance of the random 3-SAT problem, actually random 3-SAT instances have been widely used by several international SAT competitions [14–17].

Moreover, an important family of SAT instances is uniform random *k*-SAT [18]. The performance of algorithm are usually stable on random *k*-SAT instances. In last two decades, great progress has been made near the phase transition in solving random *k*-SAT. Thus, through testing SLS algorithm we can easily recognize excellent heuristics on random *k*-SAT instances, which may be beneficial for solving real-world problems [19]. Besides, it can be interesting to test SLS algorithm on solving a broad range of structured instances.

Practical algorithms for solving SAT can be mainly categorized into two main classes: 1) complete algorithms evolving out of the DPLL procedure [20] and 2) stochastic local search (SLS) algorithms based on the GSAT algorithm [21]. SLS can be regarded as a kind of heuristic search, while heuristic search is a influential and common method for solving computationally-hard combinatorial problems [22, 23]. In this paper, we are devoted to improving the performance of SLS algorithms. SLS algorithms are usually incomplete—they are able to solve a number of certain types of SAT instances very fast, especially random satisfiable instances, but cannot prove unsatisfiability [24].

In practice, a severe issue for SLS algorithms is the cycling problem, where SLS algorithms would possibly revisit candidate solutions that have been searched before [25]. This problem makes the search process of SLS algorithms stagnant, and thus results in a negative consequence on the performance of SLS algorithms. In order to deal with this serious issue effectively, Cai et al. proposed a novel configuration checking (CC) heuristic, which has achieved success in the problem of minimum vertex cover (MinVC) [26–28].

Recently, SLS algorithms achieve effectiveness in solving the maximum satisfiability (MaxSAT) problem, which is a generalization of SAT. By integrating an efficient MaxSAT local search algorithm called CCLS [15] and additive BMS (Best from Multiple Selections) [29], Chu et al. developed a new SLS algorithm for MaxSAT called CCABMS, which had a good performance on a large number of industrial MaxSAT instances [30]. Lei and Cai [31] proposed a dynamic local search algorithm, which exploited the structure of weighted partial MaxSAT (WPMS) by a carefully designed clause weighting scheme. In addition, Luo et al. presented a new SLS algorithm named CCEHC for WPMS in [22]. CCEHC employed an extended framework of CCLS with a heuristic emphasizing hard clauses, called EHC (Emphasis on Hard Clauses). Extensive experiments demonstrated that CCEHC significantly outperforms a group of SLS competitors. In the literature [32], Cai et al. proposed a novel decimation algorithm for MaxSAT, and then combined it with a local search algorithm. However, the above local search algorithms are designed specifically for solving MaxSAT, and their performance of directly solving SAT lags far behind.

When focusing on SAT solving, Cai et al. proposed a neighboring-variables-based CC heuristic to develop an SLS solver called Swcc [14] for solving SAT. Compared to the neighboring-variables-based CC heuristic, Luo et al. proposed a new clause-states-based CC heuristic called quantitative configuration checking (QCC), by combining the quantity of configurations’ variations into the CC strategy. Different from the CC strategy, where the configuration of a variable refers to the truth values of all its neighboring variables, the configuration of a variable in the QCC strategy refers to the states of all clauses it appears in the random mode rather than only in the greedy mode. Moreover the authors developed a SLS solver named Swqcc [33], which is more efficient than Swcc. However, the performance of Swqcc on random 3-SAT instances is still not satisfactory. In this situation, to enhance the efficiency of local search, Salhi proposed an aspiration mechanism and integrated it with tabu search [34]. Similarly, Cai successfully used the aspiration mechanism to improve the efficiency of the neighboring-variables-based CC heuristic for solving SAT problems [35].

In this paper, we combine the QCC heuristic and the aspiration mechanism in an effective way, and propose a new heuristic dubbed QCCA. Based on the QCCA heuristic, we also develop a new SLS solver called AspiSAT for solving SAT. Our experimental results show that the performance of AspiSAT on random 3-SAT instances exceeds that of Swqcc and Sparrow, which is an influential and effective SLS solver for SAT. Moreover, we enhance the clause weighting methods employed in both Swqcc and AspiSAT, resulting in two new SLS solvers, Ptwqcc and AspiPT, respectively. The related experiments demonstrate that Ptwqcc and AspiPT perform much better than Swqcc and AspiSAT on random 5-SAT instances.

The remainder of our paper is organized as follows. In Section 2, we provide necessary preliminaries. Section 3 presents a brief review of the stochastic local search algorithm. Section 4 shows the pattern detection heuristics. In Section 5, we combine quantitative configuration checking heuristics with aspiration mechanism. In Section 6, we propose the stochastic local search algorithm based on QCCA heuristic. In Section 7, we evaluate the performance of AspiSAT, Swqcc and Sparrow on random 3-SAT instances and structured instances. Further empirical analyzes on probability and threshold weighting method and evaluation of AspiSAT and Ptwqcc are demonstrated in Section 8. Finally, Section 9 concludes the paper and lists some future work.

## Preliminaries

Given a set of *n* Boolean variables *V* = {*x*_1_, *x*_2_, ⋯, *x*_*n*_}, and a set of 2*n* literals associated with each variable in *V*, i.e., *L* = {*x*_1_, ¬*x*_1_, *x*_2_, ¬*x*_2_, ⋯, *x*_*n*_, ¬*x*_*n*_}, a clause is a disjunction of literals. In the classic *k* − *SAT* problem, each clause consists of a fixed number of *k* literals. A propositional formula *F* can be expressed in the conjunctive normal form, i.e., *F* = *c*_1_ ∧ *c*_2_ ∧ ⋯ ∧ *c*_*m*_, where *m* is the number of clauses and *c*_*i*_(1 ⩽ *i* ⩽ *m*) is a clause in *F*. In this paper, we use the notation *V*(*F*) to represent all the variables in the formula *F*, the notation *C*(*F*) to represent all the clauses in the formula *F* and the notation *r* = *m*/*n* to denote the clause-to-variable ratio of the formula *F*. Two different variables are called neighboring variables if they appear simultaneously in at least one clause, and the notation *N*(*x*) = {*y*|*y* ∈ *V*(*F*)} (y and x are neighbor variables) is used to represent all neighboring variables of the variable *x*. We also define the notation *CL*(*x*) = {*c*|c is a clause which variable x appears} to represent the set of all clauses where the variable *x* appears.

A mapping *α*: *V*(*F*) → {*true*, *false*} is called an assignment. If *α* maps all variables to a Boolean value, then this assignment is complete. For SLS algorithms for solving SAT, a candidate solution is a complete assignment. Given a complete assignment, any clauses of formula *F* has two possible states: satisfied or unsatisfied. A clause is satisfied if and only if at least one literal of the clause is true under the assignment *α*; otherwise, the clause is unsatisfied. Given a formula *F*, an assignment *α* satisfies *F* if and only if *α* satisfies all the clauses in the formula *F*. The *SAT* problem is to decide whether there exists a complete assignment that satisfies all clauses in *F*.

Since these three SLS solvers (namely AspiSAT, Ptwqcc and AspiPT) proposed in this paper are all dynamic local search algorithms, here we give an brief introduction of related concepts of dynamic local search. In the dynamic local search, each clause *c* (*c* ∈ *C*(*F*)) in the formula *F* is associated with a positive integer (*c*) as its weight, and we use the notation weight(*c*) to denote the weight of the clause *c*. The average weight of all clauses is recorded as $\bar{w}$. We use the notation *cost*(*F*, *α*) to represent the sum of the weights of all unsatisfied clauses in the formula *F* under the assignment *α*. For any variable *x* in the formula *F*, when the variable *x* is flipped, such that the assignment *α* becomes a new assignment *β*, we define *score*(*x*) = *cost*(*F*, *α*) − *cost*(*F*, *β*). In this paper, a clause *c* is large-weighted if and only if weight(*c*) ⩾ 2.

## Stochastic local search algorithm for solving SAT problems

### The basic framework

The basic framework of the SLS algorithm for solving SAT problems is described as follows:

- **Step 1**: For the CNF formula F, the algorithm randomly generates a complete assignments to all variables in formula F;
- **Step 2**: The algorithm performs the search process in an iterative manner: in each search step (also known as iteration), the algorithm picks a variable according to a heuristic and flips the selected variable (i.e., modifying the truth value of the variable accordingly);
- **Step 3**: The algorithm will repeat Step 2 until it finds a complete assignment that satisfies the formula *F*, or the number of steps performed by the algorithm exceeds the preset step limit;
- **Step 4**: Once the search process is terminated, if the algorithm finds an assignment that satisfies F, then the algorithm reports this assignment; otherwise, it reports ‘Unknown’.

The pseudo code for a typical stochastic local search algorithm for SAT can be seen in Algorithm 1, where *α* is a variable assignment and *G* is a set of variables in the given formula *F*.

**Algorithm 1**: SLS-for-SAT

**Input**: *propositional formula F*

**Output**: *satisfying assignment of F* **or** ‘unknown’

1 **begin**

2  *α* ≔ *InitialiseSearch*(*F*)

3  **while not** *Terminate*(*F*, *α*) **do**

4   **if** *Restart*(*F*, *α*) **do**

5    *α* ≔ *ReInitialiseSearch*(*F*);

6   **else**

7    *G* ≔ *SelectVarsToFlip*(*F*, *α*);

8    *α* ≔ *FlipVars*(*F*, *α*, *G*);

9   **end**

10  **end**

11  **if** *Solved*(*F*, *α*) **then**

12   **return** *α*

13  **else**

14   **return** ‘unknown’

15  **end**

16 **end**

### Greedy mode and diversification mode

SLS algorithms for solving SAT problems usually work between two modes [36]: the greedy mode and the diversification mode. In the greedy mode, SLS algorithms prefer to flip those variables that can reduce the number of unsatisfied clauses. In the diversification mode, SLS algorithms tend to explore the search space in order to better avoid the local optimum, so a random strategy is usually employed to accomplish the task. Li et al. proposed a heuristic based on the concept of promising decreasing variable (PDV), and developed an SLS solver G^2^WSAT [37] using this PDV-based heuristic. The PDA-based heuristic has been widely used by winning SLS solvers in international SAT Competitions (e.g., G^2^WSAT [37], adaptG^2^WSAT [38], gNovelty+ [39], Sparrow [40], and EagleUP [41]). Among these solvers, Sparrow [40] achieves the best performance for solving SAT instances. Compared with the improvements in the greedy mode, recently more related work is focused on how to improve heuristics in the diversification mode. Most of the improvements for heuristics in the diversification mode belong to the Novelty family, for instance, Novelty [42], Novelty+ [43], Novelty++ [37], Novelty+p [38], and adaptNovelty+ [44]. Unlike the Novelty series, the probability distribution based heuristic [40] used by the Sparrow solver [40] is a recent breakthrough in the diversification mode.

## Review of configuration checking heuristics

As mentioned before, the cycling problem is a major bottleneck of stagnating the performance of SLS algorithms, and configuration checking (CC) is proposed for handling this problem. CC was originally proposed for improving the performance of SLS algorithms for solving the problem of minimum vertex cover. According to its generality, CC heuristics have also resulted in an SLS solver called Swcc for solving SAT. According to the experiments reported in the literature [14], the results show that the performance of Swcc on the random 3-SAT instances exceeds the winning solver called TNM [45] in random track of SAT competition, which from the practical perspective indicates that the CC heuristic is effective for dealing with the cycling problem.

The most important concept in CC heuristics is the definition of configuration. In the context of the SAT problem, the CC heuristic defines a configuration for each variable that appears in the formula *F*, which measures the circumstance information of the corresponding variable. In formal, the configuration of the variable *x* is a truth-valued vector, denoted by the notation *configuration*(*x*). Cai et al. gave the first definition of configuration where *configuration*(*x*) is composed of the truth value (i.e., true or false) of each variable in all neighboring variables of variable *x* (i.e., *N*(*x*)) [14]. The core idea of CC heuristic is to avoid any flipping variable whose configuration has not changed since the last flip of the corresponding variable [14].

In the literature [33], Luo et al. refined the definition of the configuration and gave a new definition of configuration based on the clause states. The related experiments reported in that literature showed that the performance of Swqcc with the newly defined configuration is better than Swcc which adopts the original definition of configuration. This paper is also focused on the clause-states-based configuration, so we give a formal definition of the clause-states-based configuration as follows.

**Definition 1** Given a formula *F* in CNF form and a complete assignments *a*, the configuration of the variable *x* is a vector, denoted by the notation *configuration*(*x*). The vector *configuration*(*x*) is composed of the states (i.e., satisfied or unsatisfied) of each clause in the set *CL*(*x*) consisting of all clauses where the variable *x* appears.

In [33], Luo et al. not only redefined the definition of configuration, but also quantified the changes of configuration; then Luo et al. proposed the quantitative configuration checking (QCC) heuristic. The QCC heuristic uses the notation *confvariation*(*x*) to represent the number of configuration changes since the last flip of the variable *x* [33]. For each variable *x*, the initial value of *confvariation*(*x*) is 1, and the specific update operations of *confvariation*(*x*) can be found in [33]. It is worth noting that the literature [33] also uses a smoothing mechanism for *confvariation*(*x*). In this paper, we do not smooth the *confvariation*(*x*) based on preliminary experiments.

As mentioned in [14], previous heuristics never consider the circumstance information of a variable when selecting a variable to be flipped, but take a number of variable properties (e.g., score [21], break [46] and age [47]) into account. However, the family of CC heuristics consider both the variable properties and the circumstance information of the variables when selecting a variable to be flipped. This is the essential difference between the family of CC heuristics and the previous heuristics.

## Combining quantitative configuration checking heuristics with aspiration mechanism

Although the QCC heuristic achieves improvement over the original CC heuristic, according to the implementation details described in [33], the QCC heuristic, which is similar to the original CC heuristic, still makes SLS algorithms ignore flipping a number of variables with relatively large *score*(*x*) when reaching the local optima. The aspiration mechanism is an effective way to handle this problem, and has been successfully applied to the tabu search and the original CC heuristic. The aspiration mechanism renders the CC strategy more flexible by selecting the variables with great scores to flip. Without aspiration, variables in the diversification mode may be flipped mistakenly. This would delay the local search transferring to promising regions of search space. The aspiration mechanism corrects such mistakes and thus avoids the detention [19]. In this work, we focus on combining the QCC heuristic with the aspiration mechanism in an effective way and then propose a new heuristic dubbed QCCA (Quantitative Configuration Checking with Aspiration).

In the QCCA heuristic, an important concept is the significant decreasing (SD) variable [35]. Due to its importance, we give its formal definition.

**Definition 2** Given a formula F of the CNF form and a complete set of true value assignments *α*, the variable *x* is a SD variable if and only if *score*(*x*) ⩾ *κ*, where the threshold *κ* is a relatively large integer.

In this work, when solving random 3-SAT instances, the threshold *κ* is set to the average clause weight $\bar{w}$; when solving other instances, the threshold *κ* is set to 2.

Since the QCCA heuristic is improved from the QCC heuristic, the concepts and definitions used in the QCCA heuristic are consistent with the QCC heuristic. According to the literature [33], the QCC heuristic maintains an important set of candidate variables *G*. In this paper, the QCCA heuristic maintains the candidate variable set *G* in the same way as the QCC heuristic does. Moreover, in order to be consistent with the QCC heuristic, the QCCA heuristic selects the variable to be flipped between the greedy mode and the diversification mode. The QCCA heuristic chooses which mode to select the variable to be flipped depending on whether or not the set of candidate variable *G* and the set of variable *SD* are empty. If the candidate variable set *G* or the *SD* variable set is not empty, the QCCA heuristic works in the greedy mode to select the variable; otherwise the QCCA heuristic works in the diversification mode to select the variable. The details of how the greedy mode and the diversification mode work are described as follows.

1. When the QCCA heuristic works in greedy mode, the QCCA heuristic first determines whether or not *G* is empty; if *G* is not empty, the QCCA heuristic will select the variable with the largest *score* to be flipped from the set *G*; otherwise (*G* set is empty), the QCCA heuristic will activate the aspiration mechanism, i.e., selecting the SD variable with the largest *score* to be flipped. If there is more than one variable with the largest *score*, then it will select the one with the maximum *confvariation*.
2. When the QCCA heuristic works in the diversification mode, the QCCA heuristic first randomly picks an unsatisfied clause *c*, and then selects the variable with the largest *confvariation* in the clause *c* to be flipped. If there is more than one variable with the largest *confvariation*, select the variable that has not been flipped most recently.

## Stochastic local search algorithm based on QCCA heuristic

### Smoothed clause weighting method

The clause weighting method, especially the smoothed clause weighting method, significantly improves the performance of the SLS algorithm for solving SAT instances. The smoothed clause weighting method has been adopted by many advanced SLS solvers [40, 41, 48, 51]. AspiSAT adopts the method of smoothed clause weighting(SCW) used by Swqcc, which resembles the SAPS weighting method [48].

In the SCW method, each clause has a weight, weight(c), which is a positive integer. In the initialization phase, the weight of each clause is set to 1. Whenever the SLS algorithm activates the SCW method, the weight of each unsatisfied clause will be increased by 1. In addition, the SCW method will use the smoothing mechanism periodically. The main rule used by the SCW smoothed mechanism to maintain clause weights is described as follows:

Rule 1. When the average clause weight $\bar{w}$ is greater than a fixed threshold *δ*, the weight of each clause appearing in the formula will be smoothed: for each clause *c* ∈ *C*(*F*), $weight\left(c\right)=\left\lfloor \gamma *weight\left(c\right)\right\rfloor +\left\lceil \left(1-\gamma \right)*\bar{w}\right\rceil$, where *γ* is a real-valued number ranging from 0 to 1 (0 < *γ* < 1).

In the AspiSAT algorithm, AspiSAT activates the SCW method when the algorithm reaches local optima.

### AspiSAT algorithm

In this section, we present a new SLS algorithm dubbed AspiSAT, which is based on the QCCA heuristic. We list the pseudo code of the AspiSAT algorithm in Algorithm 2, and describe the details of the AspiSAT algorithm as follows.

In the initialization phase of the algorithm, the algorithm randomly generates a complete assignment *α* as the initialization solution, and the weight of each clause is set to 1. Then for each variable *x*, the algorithm calculates the *score*(*x*) according to the initialized assignment *α*. Also, for each variable *x*, the property *confvariation*(*x*), which measures the frequency of the change on *x*’s configuration, is initialized to 1. After that, the algorithm puts all variables with *score* > 0 into *G*, the set of candidate variable. Then *G* is maintained during the search process of the algorithm (Lines 14 and 20 in Algorithm 2), and flipping the variables in *G* would decrease the total weight of all unsatisfied clauses.

When the initialization phase is complete, the algorithm performs the search process in an iterative manner: the algorithm would select and flip a variable in each search step (also known as iteration) until the algorithm finds a satisfying assignment or the number of search steps exceeds the step limit. In each search step, the algorithm selects the variable to be flipped according to the selection mechanisms employed in the greedy mode and the diversification mode of the QCCA heuristic. It is worth noting that, in the current search step, when the candidate variable set *G* and *SD* variable set are both empty, the algorithm will be considered as it reaches a local optimum, and the algorithm will activate the SCW method to update the clause weights. After the execution of the SCW method is finished, the algorithm will select a variable to be flipped in the diversification mode.

In each step, the algorithm would flip the selected variable and then update the candidate variable set *G* as well as the variable properties, such as *score*, *confvariation*.

Once the search process is finished, if the algorithm finds a satisfying assignment, the assignment reports such assignment as a solution to the formula; otherwise, the algorithm reports ‘Unknown’.

**Algorithm 2**: AspiSAT

**Input**: CNF-formula *F*, *maxSteps*

**Output**: A satisfying truth assignment *α* of *F* or *Unknown*

1 **begin**

2  initialize a random assignment *α*;

3  initialize all *weight(c)* as 1 and compute *score*(*x*) for each variable *x*;

4  initialize *confvariation*(*x*) as 1 for each variable *x*;

5  put variable with *score*(*x*) > 0 into the *G* set;

6  **for** *step* ← 1 **to** *maxSteps* **do**

7   **if** *αsatisfiesF* **then return** *α*;

8   **if** *G* ≠ *ϕ* **then**

9    *v* ← x with the greatest *score*(*x*) in *G*, breaking ties by preferring the one with the greatest *confvariation*(*x*);

10   **else**

11    **if** *there exist SD variables* **then**

12     *v* ← *x* with the SD variable with the greatest *score*(*x*), breaking ties in favor of the one with greatest *confvariation*(*x*).

13    **else**

14     increase all unsatisfied clauses’ *weights* by 1;

15     *G* ← *G* ∪ {y | *score*(*y*) > 0 & *confvariation*(*y*) > 0};

16     **if** *ω* > *δ* **then**

17      smooth clause weights by ***Rule*** *δ*;

18     *c* ← randomly choose an unsatisfied clause;

19     *v* ← the greatest *confvariation*(*x*) variable in clause *c*, breaking tie by choosing least recently flipped variable;

20   flip *v*; update *confvariation* and *score*;

21   *G* ← (*G*- {y | *score*(*y*) ⩽ 0} ∪ {y | *score*(*y*) > 0 & *y* ∈ *N(v)*};

22  **return** *Unknown*;

23 **end**

## Experimental evaluation of the AspiSAT algorithm

In this section, we first briefly describe the testing benchmarks and the experimental setups. Then, we evaluate the performance of AspiSAT, Swqcc [33], Sparrow [40] and gNovelty+GCwa [52] on random 3-SAT instances and structured instances. The experiment is divided into four parts. In the first part, we compare the performance of AspiSAT, Swqcc, Sparrow and gNovelty+GCwa on large-scale random 3-SAT instances (2500 ⩽ ♯*var* ⩽ 50000) in random track of the international SAT competition; In the second part, we compare the performance of AspiSAT, Swqcc and Sparrow on a huge-scale random 3-SAT instance (55000 ⩽ ♯*var* ⩽ 100000). In the third part, in order to show the significant performance gap between AspiSAT and Swqcc, we compare the performance of AspiSAT and Swqcc on the larger-scale random 3-SAT instance (110000 ⩽ ♯*var* ⩽ 150000). In the fourth part, we compare the performance of AspiSAT and Sattime [53] on the satisfiable structured instances; we would like to note that Sattime is well-known as it is an efficient SLS solver for solving structured SAT instances. Finally, we summarize and analyze the results of these comparative experiments.

### Testing benchmarks

In this experiment, we set up four testing benchmarks. The first benchmark contains large-scale random 3-SAT instances used in the Random SAT Track of the International SAT11 Competition [49]; The reason why we did not adopt the medium-sized random 3-SAT instances used in the SAT competition is that for modern SLS algorithms, those instances are too simple and can be quickly solved. The second and third testing benchmarks are consisting of random 3-SAT instances generated by the fixed clause length model. The second testing benchmark includes those random 3-SAT instances with 55000 ⩽ ♯*var* ⩽ 100000; the third testing benchmark contains those random 3-SAT instances with 110000 ⩽ ♯*var* ⩽ 150000.

The fourth testing benchmark is composed of selected satisfiable instances used in the Crafted Track of the international SAT11 Competition. We do not consider those unsatisfiable instances because they cannot be determined by any SLS algorithms.

All instances in the first three testing benchmarks sets share the same clause-to-variable ratio of 4.2, and they are all satisfiable. Hence these instances can be used to test the performance of the SLS algorithm.

### Competitors and experimental setup

In this experiment, the AspiSAT solver is implemented in C++. Based on preliminary experiments, for AspiSAT, the smoothing threshold *δ* is set to 200 + (♯*var* + 250)/500, and the parameters *β* and *γ* are set to 0.3. In order to make our comparison fair, the parameter settings used by the Swqcc solver [33] are the same as those ones used in the AspiSAT solver. The Sparrow solver and the Sattime solver use the version of the International SAT11 Competition [50]. It is worth noting that Sparrow [40] is one of the best-performing SLS solvers for solving random SAT instances, while Sattime [53] is one of the best performing SLS solvers for solving the structured SAT instances. The gNovelty+GCwa solver [52] is an effective local search solver for solving SAT.

All our experiments were conducted on a workstation with an Intel(R) Core(TM) i7-2620 CPU, clocked at 2.7GHz and 7.8GB of memory. The operating system of the machine is GNU/Linux.

### Evaluation criteria

In this experiment, the evaluation criteria we used were similar to those used in the International SAT Competition. We compare the average time and success rate of each algorithm in solving each set of instances. We set the cutoff time for each solver run: for the first testing benchmark, the cutoff time is set to 1000 seconds; for the second testing benchmark, the cutoff time is set to 2000 seconds; for the third testing benchmark, the cutoff time is set to 3000 seconds; for the fourth testing benchmark, the cutoff time is set to 2000 seconds.

For the first testing benchmark, each solver performs 100 runs on each instance; for the second testing benchmark, each solver performs 10 runs on each instance; for the third testing benchmark, each solver performs 1 run on each instance; for the fourth testing benchmark, each solver performs 10 runs on each instance. For each solver run, it is said to be successful if and only if the solver finds a satisfying assignment; otherwise it is said to be failed.

### Evaluation results

#### Discussion on the experimental results on the first testing benchmark

Table 1 shows the performance of AspiSAT, Swqcc, Sparrow and gNovelty+GCwa on the first testing benchmark. According to the results, in terms of the averaged run time, the performance of AspiSAT and Swqcc significantly outperform Sparrow and gNovelty+GCwa. The averaged run time of gNovelty+GCwa is even larger than 1000.0 on the 5 sets of instance sets (k3-v25000, k3-v30000, ks-v35000, ks-v40000 and k3-v50000). Similarly, in terms of the success rate, AspiSAT achieves 100% in each instance family, while Swqcc has a success rate of less than 100% on 2 sets of instance sets (k3-v40000 and k3-v50000), and the success rates of Sparrow on the 6 sets of instance sets (k3-v2500, k3-v25000, k3-v30000, k3-v35000, k3-v40000 and k3-v50000) are less than 100%. As for gNovelty+GCwa, the success rates on all the 10 sets of instance sets are less than 100%. Especially on the last instance family which is also the most difficult instance family to be solved (k3-v50000), the success rates of AspiSAT, Swqcc and Sparrow are 100%, 99.8%, 73.3% and 0.0% respectively. This shows that the performance of Sparrow and gNovelty+GCwa is significantly lower than AspiSAT and Swqcc. Based on the experimental results in Table 1, we could conclude that AspiSAT performs best on the first testing benchmark.

**Table 1:** Comparative results of AspiSAT, Swqcc, Sparrow and gNovelty+GCwa on the first benchmark.

Instance Class	gNovelty+GCwa		Sparrow		Swqcc		AspiSAT	
	♯suc	avg time	♯suc	avg time	♯suc	avg time	♯suc	avg time
k3-v2500	91.2%	129.3	98.8%	24.0	100.0%	11.5	100.0%	7.4
k3-v5000	67.7%	450.3	100.0%	13.8	100.0%	12.4	100.0%	7.5
k3-v10000	22.7%	840.4	100.0%	29.0	100.0%	21.4	100.0%	15.4
k3-v15000	0.4%	997.8	100.0%	42.1	100.0%	33.3	100.0%	22.6
k3-v20000	0.2%	998.7	100.0%	77.7	100.0%	51.9	100.0%	37.2
k3-v25000	0.0%	>1000.0	99.4%	141.6	100.0%	82.0	100.0%	55.9
k3-v30000	0.0%	>1000.0	99.0%	178.5	100.0%	104.6	100.0%	72.0
k3-v35000	0.0%	>1000.0	94.8%	315.0	100.0%	159.6	100.0%	106.2
k3-v40000	0.0%	>1000.0	93.1%	285.7	99.9%	142.2	100.0%	101.1
k3-v50000	0.0%	>1000.0	73.3%	541.5	99.8%	215.6	100.0%	184.2

♯suc means the success rate; as each solver performs 100 runs on each instances, avg time means the average time per a run.

#### Discussion on the experimental results on the second testing benchmark

We also compared the performance of AspiSAT, Swqcc and Sparrow on large-scale random 3-SAT instances (55000 ⩽ ♯*var* ⩽ 100000). The experimental results are summarized in Table 2. According to the experimental results, AspiSAT surpasses Swqcc and Sparrow in terms of both the success rate and the averaged run time. From the perspective of the success rate, AspiSAT is able to achieve the success rate higher than 98% for each instance family on the second testing benchmark (There are 10 instance families in total, among which the success rates of 6 instance families are 100%). The success rate of Swqcc is 100% on only 3 instance families, and the success rate of Swqcc is lower than that of AspiSAT on other instance families. Also, the success rates of Sparrow on these instance families range from 51.6% to 96.3%, which are significantly lower than AspiSAT and Swqcc. In particular, on the largest instance family (k3-v100000), the success rate of AspiSAT is 98.4%, while as a comparison, the success rates of Swqcc and Sparrow are only 91.4% and 51.6%, respectively.

**Table 2:** Comparative results of AspiSAT, Swqcc and Sparrow on the second benchmark.

Instance Class	Sparrow		Swqcc		AspiSAT	
	♯suc	avg time	♯suc	avg time	♯suc	avg time
k3-v55000	96.3%	573.6	100.0%	201.1	100.0%	171.5
k3-v60000	94.5%	652.8	100.0%	219.5	100.0%	191.9
k3-v65000	90.2%	852.1	100.0%	276.0	100.0%	236.8
k3-v70000	89.6%	844.4	99.7%	309.7	99.8%	267.7
k3-v75000	81.4%	1077.5	99.8%	408.0	100.0%	342.0
k3-v80000	77.3%	1134.8	99.3%	480.6	99.9%	377.3
k3-v85000	69.0%	1290.7	99.1%	620.7	100.0%	441.2
k3-v90000	63.8%	1408.8	98.9%	662.5	100.0%	516.2
k3-v95000	50.9%	1585.3	96.2%	847.8	99.4%	623.3
k3-v100000	51.6%	1556.4	91.4%	967.8	98.4%	693.4

♯suc means the success rate; as each solver performs 100 runs on each instances, avg time means the average time per a run.

In addition, in terms of averaged run time, AspiSAT uses less than 700 seconds on all instance families. The average run time of Swqcc on all instance families ranges from 201.1 to 967.8, while that of Sparrow ranges from 573.6 to 1583.6. In particular, on the k3-v100000 instance family, the averaged run time of AspiSAT is 693.4 seconds, while the averaged run time of Swqcc and Sparrow is 967.8 seconds and 1556.4 seconds, respectively. The experimental results show that AspiSAT performs best on the second testing benchmark.

#### Discussion on the experimental results on the third testing benchmark

In order to better illustrate the performance gap between AspiSAT and Swqcc and the performance gap between the QCCA heuristic and the original QCC heuristic, we further explore the performance of our AspiSAT solver and the Swqcc solver on a larger-scale random 3-SAT instances (110000 ⩽ ♯*var* ⩽ 150000). Table 3 reports the experimental results of this empirical comparison and indicates that AspiSAT performs clearly better than Swqcc.

**Table 3:** Comparative results of AspiSAT and Swqcc on the third benchmark.

Instance Class	Swqcc		AspiSAT	
	♯suc	avg time	♯suc	avg time
k3-v110000	96.0%	1335.0	99.0%	921.4
k3-v120000	93.0%	1500.6	100.0%	1068.7
k3-v130000	80.0%	2059.2	100.0%	1345.6
k3-v140000	70.0%	2199.0	96.0%	1631.7
k3-v150000	72.0%	2231.4	89.0%	1867.3

♯suc means the success rate; as each solver performs 100 runs on each instances, avg time means the average time per a run.

According to the experimental results, the performance gap between AspiSAT and Swqcc in terms of both the success rate and the average run time shows that the improvement of the QCCA heuristic over the original QCC heuristic is substantial, which indicates the effectiveness of the aspiration mechanism. We would like to note that the AspiSAT solver could also be combined with the probability distribution heuristic adopted by the Sparrow solver, and the performance might be further improved.

#### Discussion on the experimental results on the fourth testing benchmark

It is widely recognized that, for solving the structured SAT instances, complete solvers based on conflict driven clause learning (CDCL) shows the best performance. However, it is also very interesting to test the performance of SLS solvers on solving structured SAT instances, and Sattime is an efficient SLS solver for solving structured SAT instances.

To demonstrate the robustness of the AspiSAT solver, we compare the performance of AspiSAT, Swqcc and Sattime on the fourth testing benchmark. According to the experimental results reported in Table 4, it is clear that AspiSAT achieves the comparable performance compared to Sattime on the fourth testing benchmark, and even performs better than Sattime on a number of instance families. When focusing on the comparison between AspiSAT and Swqcc, the AspiSAT solver performs better or achieves the comparable performance compared to Swqcc. More particularly, out of 7 instance families in total, AspiSAT performs better than Swqcc in terms of the success rate on 3 instance families, and achieves the same success rate compared to Swqcc on the other 4 instance families. The experimental results show that the QCCA heuristic is able to achieve improvements over the original QCC heuristic on solving structured SAT instances.

**Table 4:** Comparative results of AspiSAT, Swqcc and Sattime on the fourth benchmark.

Instance Class	♯instances	Sattime		Swqcc		AspiSAT	
		♯suc	avg time	♯suc	avg time	♯suc	avg time
289	15	100.0%	0.06	100.0%	<0.01	100.0%	<0.01
automata-synchronization	7	0.0%	>2000.0	7.1%	1926.6	14.3%	1830.3
battleship	14	100.0%	14.1	100.0%	<0.01	100.0%	<0.01
Green Tao	3	100.0%	32.3	93.3%	236.0	96.7%	201.6
sgen	10	97.0%	137.5	40.0%	1325.5	40.0%	1342.2
SRHD-SGI	28	53.2%	1023.1	43.5%	1226.8	45.0%	1176.9
VanDerWaerden_pd_3k	7	77.1%	679.1	100.0%	45.4	100.0%	85.4

♯suc means the success rate; as each solver performs 100 runs on each instances, avg time means the average time per a run.

We would like to note that Sattime adopts reasoning mechanisms before local search in solving structured SAT instances. However, in order to better demonstrate the effect of QCCA heuristic, we do not integrate reasoning mechanisms into the implementation of AspiSAT. By combining effective reasoning mechanisms, the performance of AspiSAT could be enhanced on solving structured SAT instances.

**Summary**: The above experimental results show that AspiSAT performs better than Swqcc, Sparrow and gNovelty+GCwa on solving random 3-SAT instances, which indicates the effectiveness of the QCCA heuristic. Moreover, the QCCA heuristic achieves improvement over the original QCC heuristic on solving both random 3-SAT instances and structured SAT instances.

## Improving clause weighting method for AspiSAT solver and Swqcc solver

In this section, by improving the original SCW weighting method of AspiSAT solver and Swqcc solver, we can develop new SLS solvers based on AspiSAT and Swqcc, respectively: AspiPT and Ptwqcc, which shows performance improvement on solving random 5-SAT instances. Our experimental results show that AspiPT and Ptwqcc perform significantly better than AspiSAT and Swqcc on random 5-SAT instances, and the performance of AspiPT and Ptwqcc on random 7-SAT instances is highly comparable to that of AspiSAT and Swqcc.

### Probability and threshold weighting method

In this subsection, we present a probability and threshold weighting (PTW) method which is similar to the PAWS weighting method [51]. The PTW weighting method has a total of two components: an increasing weight component and a decreasing weight component. In the search phase, the original PAWS method would activate the decreasing weight component in a fixed period. Unlike the PAWS method, the PTW weighting method activates the weight decreasing component in a probabilistic manner—whenever PTW is activated, PTW determines which component to be called depending on the probability parameter *sp* and the threshold *θ*.

The PTW weighting method will be executed as follows. First, a probability *p* is randomized. When *p* < *sp* and the number of large weight clauses is greater than *θ*, the PTW performs the decreasing weight component: for each large weight clause, the clause weight is decreased by 1; otherwise, the PTW executes the increasing weight component: for each unsatisfied clause, the clause weight is increased by 1.

We replace the original SCW weighting method with the proposed PTW weighting method in AspiSAT and Swqcc, resulting in new SLS solvers called AspiPT and Ptwqcc, respectively.

### Empirical evaluation of AspiPT and Ptwqcc

In this section, we evaluate the performance of AspiPT, Ptwqcc, AspiSAT and Swqcc on random 5-SAT instances and random 7-SAT instances. The random 5-SAT testing benchmark we used contains all large-scale random 5-SAT instances in the International SAT competition (10 instances per instance family). The random 7-SAT testing benchmark we used includes random 7-SAT instances with ♯*var* = 150 and ♯*var* = 200 in the International SAT competition (10 instances per instance family).

Then we introduce the parameter settings for AspiPT and Ptwqcc. For solving random 5-SAT instances, the probability parameter *sp* in AspiPT and Ptwqcc is set to 0.75 and the threshold *θ* is set to 8. For solving random 7-SAT instances, the probability parameter *sp* in AspiPT and Ptwqcc is set to 0.9 and the threshold *θ* is set to 10. In addition, since AspiPT uses the aspiration mechanism, we set the parameter *κ* of AspiPT to 2.

In our experiment, each solver performs 10 runs on each instance. For each solver run, the cutoff time is set to 1000 seconds.

Table 5 reports the experimental results of AspiPT, Ptwqcc, AspiSAT and Swqcc on random 5-SAT instances and random 7-SAT instances. As can be seen from the experimental results, the performance of AspiPT and Ptwqcc on random 5-SAT instances is significantly better than that of AspiSAT and Swqcc. We would like to note that Swqcc and AspiSAT cannot solve random 5-SAT instances with ♯*var* ⩾ 1250 within the cutoff time, while AspiPT and Ptwqcc can at least solve random 5-SAT instances with ♯*var* = 2000. In addition, the performance of AspiPT and Ptwqcc is highly comparable to that AspiSAT and Swqcc on solving random 7-SAT instances. The experimental results show that the QCCA heuristic and the QCC heuristic can cooperate well with different clause weighting methods (SCW method and PTW method).

**Table 5:** Comparative results of AspiPT, Ptwqcc, AspiSAT and Swqcc on random 5-SAT instances and random 7-SAT instances.

Instance Class	Swqcc		Ptwqcc		AspiSAT		AspiPT	
	♯suc	avg time	♯suc	avg time	♯suc	avg time	♯suc	avg time
k5-v750	55.0%	672.8	95.0%	147.4	96.0%	677.5	95.0%	135.3
k5-v1000	1.0%	994.8	94.0%	237.1	2.0%	991.3	97.0%	238.6
k5-v1250	0.0%	>1000.0	86.0%	359.2	0.0%	>1000.0	92.0%	292.4
k5-v1500	0.0%	>1000.0	54.0%	675.6	0.0%	>1000.0	65.0%	612.2
k5-v2000	0.0%	>1000.0	20.0%	868.1	0.0%	>1000.0	19.0%	881.1
k7-v150	46.0%	731.8	40.0%	782.2	49.0%	722.0	37.0%	803.3
k7-v200	2.0%	981.4	2.0%	986.6	1.0%	990.2	3.0%	988.7

♯suc means the success rate; as each solver performs 100 runs on each instances, avg time means the average time per a run.

## Conclusion

In this paper, we propose a new QCCA heuristic, which effectively combines the QCC heuristic and the aspiration mechanism. The QCCA heuristic is able to improve the performance of the SLS algorithm for solving the SAT problem. Based on QCCA heuristics, we develop a new SLS solver AspiSAT for solving SAT problems. Extensive experiments present that the AspiSAT solver performs better than Swqcc, Sparrow and gNovelty+GCwa on solving a broad range of random 3-SAT instances. Moreover, for solving the structured SAT instances, AspiSAT outperforms Swqcc in terms of the success rate, and the performance of AspiSAT is comparable to that of Sattime.

Furthermore, we replace the original weighting method SCW in AspiSAT and Swqcc with the PTW weighting method, resulting in two new SLS solvers dubbed AspiPT and Ptwqcc, respectively. Our experiments show that AspiPT and Ptwqcc perform significantly better than AspiSAT and Swqcc on random 5-SAT instances. The conclusion shows that the QCCA heuristic and the QCC heuristic are able to cooperate well with different clause weighting methods.

For future work, we plan to integrate the probability distribution heuristic into AspiSAT, in order to further improve the performance. We would also like to redefine the concept of configuration, in order to propose new and more effective heuristics based on the QCC heuristic and the QCCA heuristic for solving other types of SAT instances.
